# Load-bearing capacities of occlusal veneers made of all-ceramic and resin composite: do the substrate and material type affect mechanical durability?

**DOI:** 10.1186/s12903-026-09356-6

**Published:** 2026-07-27

**Authors:** Arta Lamallari, Vera Colombo, Luiza Freitas Brum Souza, Enkelejd Angelo Cankja, Abdülhakim Alpaslan Sener, Mutlu Özcan

**Affiliations:** Center for Dental Medicine, Clinic of Masticatory Disorders and Dental Biomaterials, University of Zurich, Plattenstrasse 11, CH-8032, Zurich, Switzerland

**Keywords:** Adhesion, Computer-aided design, Computer-aided manufacturing, Dental materials, Fatigue, Lithium disilicate, Prosthetic dentistry, Occlusal dental veneer, Resin composite, Zirconia

## Abstract

**Background:**

The aim of this study was to evaluate the load-bearing capacity of zirconia, lithium disilicate, and resin-matrix composite occlusal veneers bonded to different substrates: dentin surrounded by enamel (DE), dentin (D), and resin composite (C), simulating eroded teeth.

**Methods:**

Occlusal veneers (1.0 mm thick) were milled from zirconia (IPS e.max ZirCAD), lithium disilicate (IPS e.max CAD; Initial LiSi Block), and resin-matrix composite (Lava Ultimate), and bonded to the different substrates (*n* = 10 per group). Specimens were cemented, subjected to thermo-mechanical aging (1,200,000 cycles; 5–50 °C), and tested under monotonic load-to-fracture. Maximum fracture load (Fmax) data were analyzed using the Shapiro–Wilk and Levene tests for normality and homoscedasticity, followed by two-way ANOVA and Tukey’s post hoc test (α = 0.05). Weibull analysis was performed to assess structural reliability, and Pearson’s correlation analysis was used to evaluate the relationship between bonded surface area and Fmax.

**Results:**

The results indicated that both restorative material and substrate type significantly affected Fmax. Lithium disilicate veneers exhibited substrate-dependent behavior, with higher fracture loads when bonded to resin composite, intermediate values when bonded to DE, and the lowest values when bonded to D. The resin-matrix composite showed a more homogeneous response across substrates, although Fmax decreased from resin composite to dentin. Zirconia restorations demonstrated consistently high fracture loads across all substrates, without a substrate-dependent trend. Failure modes were predominantly adhesive or mixed for lithium disilicate, whereas cohesive veneer failures predominated for zirconia.

**Conclusions:**

Both restorative material and substrate condition significantly affected the strength and reliability of ultra-thin occlusal veneers. Zirconia showed the most robust performance and the lowest sensitivity to substrate variations, whereas bonding to resin composite substrates improved reliability compared with dentin. These findings highlight the importance of substrate management and adhesive optimization when restoring eroded teeth.

## Background

Tooth wear is a multifactorial condition arising from an interaction of erosion, attrition, and abrasion [1]. Consequences of dental hard tissue loss can include impaired aesthetics, loss of occlusal vertical dimension, increased sensitivity, pain, and restricted function or mastication [2]. The prevalence of tooth wear in young European adults is nearly 30%, as reported by Bartlett et al. [3]. Several direct and indirect restorative treatment options and materials can be used to substitute lost tooth substance [4–6].

Preparing teeth for full crown restorations can entail removing a large amount of tooth structure, up to 75% [7]. Tooth preparation should be kept as conservative as possible in order to preserve biological integrity and reduce the risk of adverse effects such as loss of pulp vitality [7]. With the aim of preventing excessive removal of dental hard tissue during treatment, more substance-conserving approaches have been investigated and are increasingly recommended for clinical application [7]. Minimally invasive concepts using non-retentive preparation designs and primarily relying on adhesive reconstructions, such as partial crowns or occlusal veneers, have been extensively investigated [8–13]. According to Yli-Urpo et al. [14], the implementation of a circumferential chamfer preparation for occlusal veneers appears to have no significant effect on the fracture strength of the restored tooth [14].

All-ceramic materials are well established and offer notable versatility for posterior applications [4]. Several in vitro studies have demonstrated the suitability of zirconia and lithium disilicate for minimally invasive occlusal restorations [8–13, 15]. Compared with glass-ceramics, zirconium oxide-based ceramics demonstrate superior mechanical strength [16, 17]. Studies have shown that both CAD/CAM-milled lithium disilicate (IPS e.max CAD) and zirconia (IPS e.max ZirCAD MT) occlusal veneers exhibit sufficient fatigue and fracture resistance for clinical use [10, 15]. Computer-aided design and manufacturing (CAD/CAM), particularly direct milling, has optimized the workflow for ultra-thin all-ceramic restorations by reducing time and costs while providing favourable physical properties [18].

In addition to all-ceramic materials, indirect resin-matrix composites are gaining popularity for the replacement of missing tooth structure [19, 20]. Such indirect composite materials are characterized by a hybrid composition of an organic polymer matrix and ceramic fillers [20]. Compared with all-ceramic materials such as lithium disilicate, they exhibit higher elasticity and lower brittleness [19]. A key benefit is their elastic modulus, which is considerably closer to that of dentin than that of ceramics, potentially reducing the risk of fracture [19, 21]. In addition, resin composites allow for easier intraoral repair [19] and more efficient CAD/CAM processing [20]. Literature suggests that resin-matrix composites may exhibit higher fatigue resistance than lithium disilicate in occlusal veneers [22, 23]. However, despite the reported advantages, limitations have been highlighted regarding long-term fatigue/fracture behavior and aging-related stability [24].

It is commonly known that adhesion to enamel is superior to adhesion to dentin [25]. Based on the findings of Ma et al. [25], minimal-thickness lithium disilicate restorations exhibit approximately 75% of zirconia’s load-bearing capacity when bonded to enamel, whereas lithium disilicate bonded to dentin achieves slightly more than 50% of the load-bearing capacity of zirconia [25]. Previous studies evaluating occlusal veneers bonded to enamel and/or dentin have reported load-bearing capacities above normal bite forces, indicating that these differences may not be clinically significant [8–13]. However, there are no available data providing recommendations regarding the appropriate quantity of enamel required. Furthermore, there is a lack of literature comparing enamel and dentin with composite resin as an additional substrate bonded to minimally invasive reconstructions.

The objective of this study was to investigate the load-bearing capacity of occlusal veneers made of zirconia, lithium disilicate ceramic, and resin composite when bonded to dentin or resin composite, also considering the amount of enamel surrounding the preparation. This may support material selection for restoring eroded teeth in the presence of dentin, dentin surrounded by enamel, and restorations bonded exclusively to resin composite. The null hypotheses of this study were that neither (1) the substrate type nor (2) the restorative material would affect the load-bearing capacity of ultra-thin occlusal veneers.

## Methods

### Materials and study design

**Fig. 1:**
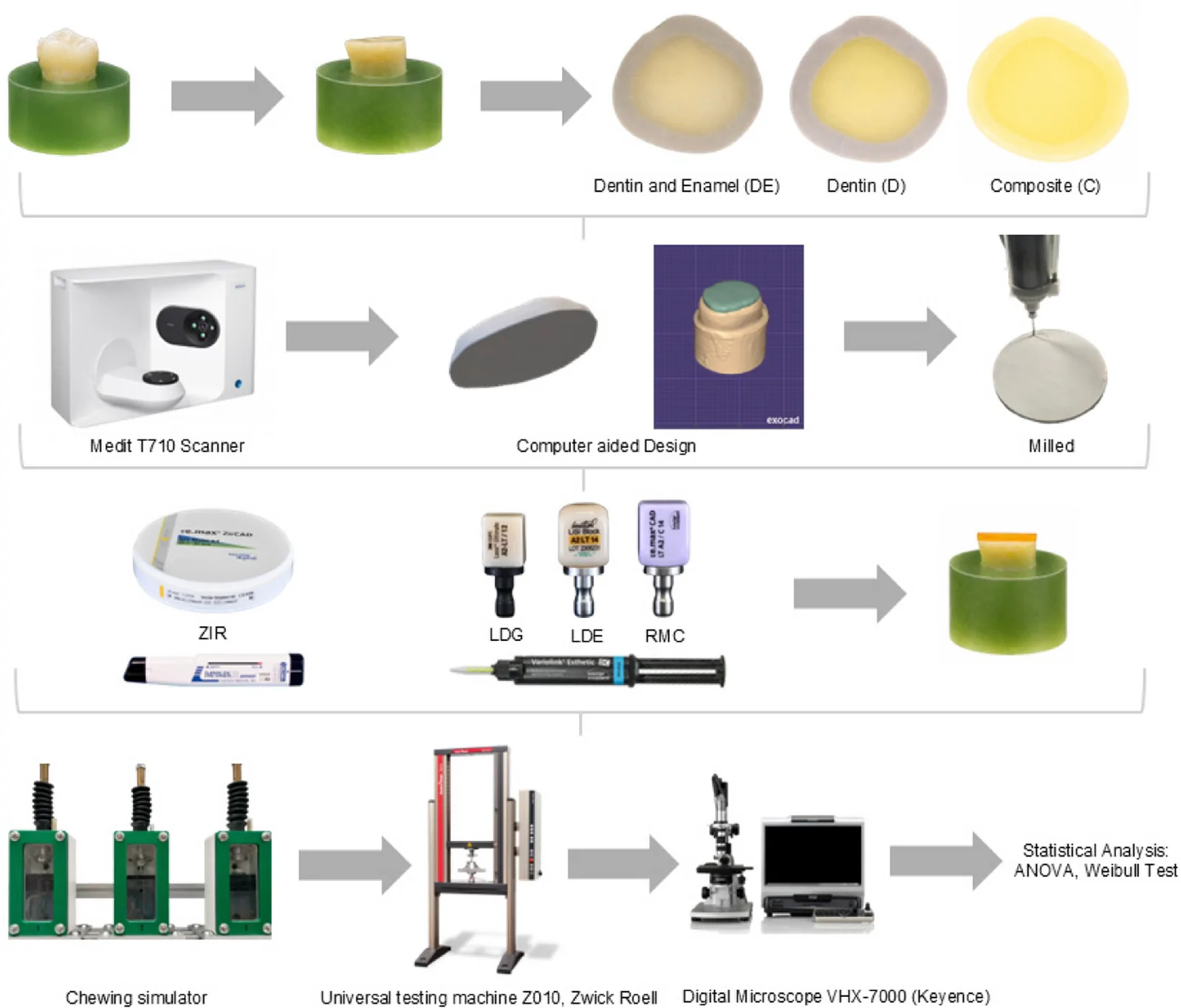
Experimental workflow illustrating substrate preparation, digital design and milling of occlusal veneers, adhesive cementation, thermo-mechanical aging, fracture testing, and failure analysis

Detailed information about the materials used in this study is provided in Table 1. This study employed a 4 × 3 factorial design (Table 2) to investigate the effects of material type (lithium disilicate IPS e.max CAD (LDE), lithium disilicate Initial LiSi Block (LDG), resin-matrix composite Lava Ultimate (RMC), and zirconia IPS e.max ZirCAD MT Multi (ZIR) and bonded substrate condition (dentin (D), dentin surrounded by enamel (DE), and resin composite buildup (C) on the mechanical performance of ultra-thin occlusal veneers (Fig. 1). A total of 120 extracted human third molars were distributed across the experimental groups, resulting in 10 specimens per material-substrate combination.

**Table 1:** Overview of the materials used in this study

Material	Manufacturer	Chemical Composition	Batch number
IPS e.max CAD	Ivoclar AG, Schaan, Liechtenstein	Glass-ceramic: matrix of glassy phase with 40–45% Li₂Si₂O₅ crystals (lithium disilicate), residual glassy matrix, minor oxides (Al₂O₃, K₂O, P₂O₅)	Y32021
IPS e.max ZirCAD, MT Multi A2	Ivoclar AG	ZrO_2_ (86.0–93.5 wg%) Y_2_O_3_ (6.5% − 8.0 wg%) HfO_2_ (≤ 5.0 wg%) Al_2_O_3_ (≤ 1.0 wg%) and other oxides (≤ 1.0 wg%)	Z064XF
Lava Ultimate	3 M Schweiz GmbH, Rüschlikon, Schweiz	Organic phase: Bis-GMA, Bis-EMA, TEGDMA, UDMA; Inorganic phase: silica (20 nm) and zirconia (4–11 nm) fillers, zirconia-silica clusters (0.6–10 μm); 79 wt% loading	10579304
Initial LiSi Block	GC Europe, Leuven, Belgium	Lithium silicate glass-ceramic: ~55–80 wt% SiO₂, ~ 10–30 wt% Li₂O, other oxides including Al₂O₃, K₂O, P₂O₅, ZnO (< 5 wt%)	2308231
Korox	BEGO, Bremen, Germany	50 μm alumina particles	L39959
Ultraetch	Ultradent Products Inc., Utah, USA	35 wt% phosphoric acid in aqueous solution	BWNV2
Ivoclean	Ivoclar AG	Alkaline zirconium oxide-based cleaning paste suspended in aqueous medium; free of phosphates	Z064KL Z05PCV
Monobond Plus	Ivoclar AG	Ethanol (50–100 wt%), methacrylated phosphoric acid ester (< 2.5 wt%), silane methacrylate compounds	Z05Z5Y
Heliobond	Ivoclar AG	bis-GMA (~ 60 wt%), TEGDMA (~ 40 wt%)	V14836
Syntac Primer	Ivoclar AG	Maleic acid (~ 4 wt%), triethylene glycol dimethacrylate (TEGDMA), water, acetone	Z05MM0
Syntac Adhesive	Ivoclar AG	Water, poly(ethylene glycol) dimethacrylate (PEGDMA), glutaraldehyde	Z05CX5
Clearfil Ceramic Primer Plus	Kuraray Noritake Dental Inc. Okayama, Japan	Silane coupling agent, 10-MDP (10-methacryloyloxydecyl dihydrogen phosphate), ethanol	2K0108
IPS Ceramic Etching Gel	Ivoclar AG	5 wt% hydrofluoric acid in gel formulation	Z05KFK
ED Primer A	Kuraray Noritake Dental Inc.	HEMA, 10-MDP, water, ethanol	910070
ED Primer B	Kuraray Noritake Dental Inc.	Aromatic tertiary amine, water, ethanol	910014
Panavia 21	Kuraray Noritake Dental Inc.	Catalyst paste: 10-methacryloyloxydecyl dihydrogen phosphate (10-MDP), hydrophobic aromatic dimethacrylate, hydrophobic aliphatic dimethacrylate, silanated silica filler, colloidal silica, catalysts. Universal paste: hydrophobic aromatic and aliphatic dimethacrylates, silanated titanium oxide and barium glass fillers, catalysts, accelerators, pigments	000088
Clearfil AP-X	Kuraray Noritake Dental Inc.	Organic matrix based on dimethacrylate monomers; inorganic filler loading ~ 86 wt%, including barium glass (radiopaque fillers)	CL0154
Variolink esthetic	Ivoclar AG	Organic matrix: UDMA-based methacrylate monomers; inorganic fillers (~ 38 wt%) including ytterbium trifluoride (YbF₃) and mixed oxide fillers; particle size approximately 0.04–0.2 μm (mean ~ 0.1 μm)	Z05KPF, Z06510
Oxyguard II	Kuraray Noritake Dental Inc.	Oxygen-inhibiting gel. Glycerol (50–70 wt%), polyethylene glycol, catalysts, accelerators, dyes	A30119 820118

**Table 2:** Experimental group classifications based on bonding substrates and restorative materials

Group	Bonding substrate	Restorative material
LDE-DE	Dentin and Enamel (DE)	IPS e.max CAD, Ivoclar AG, Schaan, Liechtenstein
LDE-D	Dentin (D)	
LDE-C	Composite (C)	
LDG-DE	Dentin and Enamel (DE)	Initial LiSi Block, GC Europe, Leuven, Belgium
LDG-D	Dentin (D)	
LDG-C	Composite (C)	
RMC-DE	Dentin and Enamel (DE)	Lava Ultimate; 3 M Schweiz GmbH, Rüschlikon, Schweiz
RMC-D	Dentin (D)	
RMC-C	Composite (C)	
ZIR-DE	Dentin and Enamel (DE)	IPS e.max ZirCAD, MT Multi A2, Ivoclar AG, Schaan, Liechtenstein
ZIR-D	Dentin (D)	
ZIR-C	Composite (C)	

**Fig. 2:**
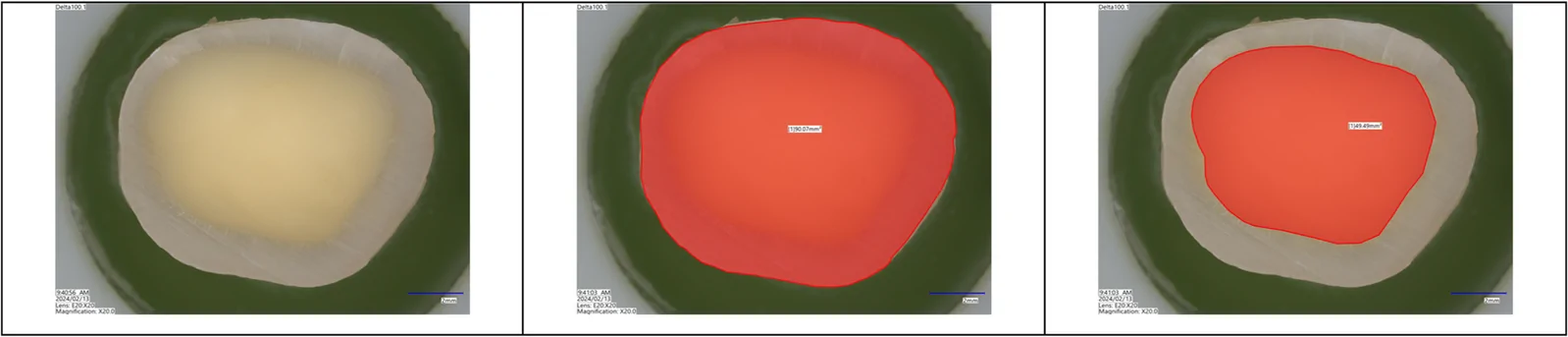
Microscopic measurements of the total and dentin surface using a VHX-7000 digital microscope at 20× magnification

For the dentin substrate (D), restorations were fabricated and bonded exclusively within the dentin borders, without including a circumferential enamel, whereas for the DE substrate a surrounding enamel margin was preserved (Fig. 2). All occlusal veneers were digitally designed, fabricated by CAD/CAM milling, and bonded using material-specific adhesive protocols. After thermo-mechanical aging, specimens were subjected to a monotonic load-to-fracture test, recording the initial (F_initial_) and maximum fracture load (F_max_). Failure modes and reliability characteristics were additionally assessed.

### Tooth preparation

For this study, extracted intact human third molars (*N* = 120) were collected. This study did not involve living human participants or any intervention on human subjects. The study used extracted human teeth obtained from the Center for Dental Medicine at the University of Zurich (Zurich, Switzerland). The teeth were extracted for clinical reasons unrelated to this study and were made available for research and other applications only after written informed consent for secondary use had been obtained from the donors, in accordance with the Swiss Federal Ordinance on Human Research [26]. All specimens were irreversibly anonymized before use in accordance with Swiss federal and cantonal regulations [26, 27]. Under applicable Swiss Human Research legislation and the guidance of the Kantonale Ethikkommission Zürich (Cantonal Ethics Committee Zurich), formal ethics committee approval was not required for this in vitro study using irreversibly anonymized extracted human teeth [27]. The study was conducted in accordance with the ethical principles of the Declaration of Helsinki [28]. After extraction, the teeth were stored in a 0.5% chloramine-T solution at 5 °C until use.

The teeth were then embedded in an auto-polymerizing resin (Technovit 4071, Kulzer, Wasserburg, Germany), with the crown exposed above the acrylic cylinder. To simulate tooth substance loss, all specimens were occlusally prepared with extension into dentin using a diamond cut-off wheel (MOD10, Struers GmbH, Copenhagen, Denmark) mounted on a low-speed precision saw (ISOMET, Buehler, Lake Bluff, IL, USA). The preparations were performed by removing the occlusal enamel until dentin exposure, resulting in sharp and well-defined margins. The exposed dentin remained surrounded by a circumferential enamel rim, which was measured using a digital microscope (VHX-7000, Keyence, Osaka, Japan).

One third of the specimens received an additional occlusal buildup with resin composite (Clearfil AP-X, shade A2; Kuraray Noritake Dental Inc., Okayama, Japan). Prior to the buildup, the enamel and dentin surfaces were conditioned with 35% phosphoric acid (Ultra-Etch, Ultradent Products Inc., Utah, USA), followed by the application of an adhesive system (Heliobond, Ivoclar AG, Schaan, Liechtenstein) and Syntac Primer and Adhesive (Ivoclar AG), according to the manufacturer’s instructions. The composite buildup was performed by a single trained operator using a standardized single increment for all specimens to ensure consistency. After polymerization, the final thickness of the composite buildup ranged from approximately 1.5 to 2.0 mm.

### Scan and computer-aided design

All specimens were digitized using a laboratory scanner (Medit T710, Seoul, South Korea). The resulting datasets were exported as STL files and imported into a dental CAD software (exocad DentalCAD, exocad GmbH, Darmstadt, Germany). Occlusal veneers were designed with a flat occlusal morphology and a standardized thickness of 1.0 mm. A uniform cement space of 40 μm, based on parameters previously adopted in CAD/CAM studies, was defined. Although the restorations presented a flat occlusal design, the preparation margin consisted of a 0.05 mm inclined segment at 60°, associated with a 0.01 mm horizontal extension and without a vertical component. This marginal configuration enabled stable and reproducible seating of each restoration, minimizing positional variations and ensuring standardized cement space thickness among specimens (Fig. 1).

### Occlusal veneer manufacturing

The restorations were fabricated from four CAD/CAM materials: LDE: lithium disilicate glass-ceramic (IPS e.max CAD, Ivoclar AG), LDG: lithium disilicate glass-ceramic (Initial LiSi Block, GC Corp., Tokyo, Japan), RMC: resin-matrix composite (Lava Ultimate, 3 M ESPE, St. Paul, MN, USA), and ZIR: zirconia (IPS e.max ZirCAD MT Multi A2, Ivoclar AG). All restorations from the LDE, LDG, RMC, and ZIR groups were milled from prefabricated blocks/discs using a 5-axis milling machine (PrograMill PM7, Ivoclar AG). Subsequently, the LDE specimens were fully crystallized in a ceramic furnace (Programat P310, Ivoclar AG), and the ZIR restorations were sintered to full density in a sintering furnace (Programat S2, Ivoclar AG) according to the manufacturer`s recommendations.

### Cementation

Restorations were bonded to the prepared teeth according to the manufacturers’ instructions for each luting system.

For the ZIR group, the prepared enamel and dentin surfaces were etched with 35% phosphoric acid (Ultra-Etch, Ultradent Products Inc., South Jordan, UT, USA) for 30 s (enamel) and 15 s (dentin), rinsed with water, and gently air-dried. ED Primer A and B were mixed at a 1:1 ratio (Kuraray Noritake Dental Inc.) and applied to enamel and dentin for 60 s, followed by gentle air-drying. The resin composite substrate was air-abraded with 50 μm alumina particles (Korox, BEGO, Bremen, Germany) at 2.8 bar for 5 s, rinsed with water spray, and gently air-dried. Clearfil Ceramic Primer Plus (Kuraray Noritake Dental Inc.) was then applied for 60 s and gently air-dried. Prior to cementation, the intaglio surface of the zirconia restorations was cleaned with a cleaning paste (Ivoclean, Ivoclar AG) for 30 s, rinsed with water spray, and gently air-dried. The intaglio surface was then air-abraded with 50 μm alumina particles at 2.8 bar for 20 s, rinsed with water spray, and gently air-dried. Clearfil Ceramic Primer Plus (Kuraray Noritake Dental Inc.) was applied for 60 s and gently air-dried. The resin cement was mixed at a 1:1 ratio (Panavia 21, Kuraray Noritake Dental Inc.) and applied prior to seating the restoration. Excess cement was carefully removed, and glycerin gel (Oxyguard, Kuraray Noritake Dental Inc.) was applied to the restoration margins for 7 min, followed by water spray cleaning.

For the LDE and LDG groups, the prepared enamel and dentin surfaces were etched and cleaned as described above. Syntac Primer (Ivoclar AG) was applied for 15 s, followed by Syntac Adhesive (Ivoclar AG) for 15 s, both on enamel and dentin. Adhesive (Heliobond, Ivoclar AG) was then applied, gently air-dried, and photo-polymerized for 10 s using a LED curing unit. Resin composite substrate was air-abraded and cleaned as described above. A silane coupling agent (Monobond Plus, Ivoclar AG) was applied for 60 s and gently air-dried. Subsequently, adhesive resin (Heliobond, Ivoclar AG) was applied for 20 s, gently air-dried, and photo-polymerized for 10 s. Prior to cementation, the intaglio surface of the lithium disilicate veneers was cleaned with a cleaning paste (Ivoclean, Ivoclar AG) for 30 s, followed by water spray rinsing and gentle air-drying. The bonding surface was etched with 5% hydrofluoric acid (IPS Ceramic Etching Gel, Ivoclar AG) for 20 s, rinsed with water spray, and gently air-dried. Monobond Plus (Ivoclar AG) was applied for 60 s and gently air-dried. Finally, the resin cement was applied (Variolink Esthetic DC, Ivoclar AG), excess cement was removed, and each surface was photo-polymerized for 20 s.

For the RMC group, the substrates were prepared as described for LDE e LDG groups. Prior to cementation, the intaglio surface of the resin composite veneers was air-abraded with 50 μm alumina particles (Korox, BEGO) at 2.8 bar for 5 s, rinsed with water spray, and gently air-dried. Monobond Plus (Ivoclar AG) was applied for 60 s and gently air-dried. Finally, the resin cement was applied (Variolink Esthetic DC, Ivoclar AG), excess cement was removed, and each surface was photo-polymerized for 20 s.

### Thermo-mechanical aging

All specimens underwent thermo-mechanical aging using a custom-made chewing simulator. The aging protocol consisted of 1,200,000 chewing cycles at a load of 49 N and a frequency of 1.67 Hz, performed under distilled water with alternating temperatures between 5 °C and 50 °C, with temperature changes every 120 s. The load was applied perpendicular to the occlusal plane using a stainless-steel indenter with a rounded tip (8 mm diameter).

### Monotonic load-to-fracture test

After confirming the integrity of the restorations, the specimens were subjected to a monotonic load-to-fracture test to determine the initial and maximum fracture load (*Finitial* and *Fmax*). *Finitial* was defined as the force corresponding to the onset of the first fracture event, as automatically detected and recorded by the software of the universal testing machine (Zwick/Roell Z050, Zwick, Ulm, Germany), while *Fmax* corresponded to the maximum load sustained before catastrophic failure. Compressive load was applied perpendicular to the occlusal surface at a crosshead speed of 1 mm/min.

After fracture, failure modes were assessed under a digital microscope (VHX-7000, Keyence, Osaka, Japan) at 20× magnification and documented using digital photographs. Failures were categorized as either substrate failure or occlusal veneer failure. For substrate failures, the classification was based on the amount of cement and/or restorative material remaining adhered to the substrate surface after failure. Type 0 corresponded to adhesive failure, with no cement or restorative material remaining on the substrate. Type 1 and Type 2 corresponded to mixed failures, with less than one-third and more than two-thirds of the substrate surface covered by cement and/or restorative material remnants, respectively. Type 3 corresponded to complete coverage of the substrate surface by cement and/or restorative material remnants after failure. Occlusal veneer failures were classified independently as Type A (debonding without fracture of the restoration) or Type B (cohesive fracture within the restorative material).

### Statistical analysis

Statistical analyses were performed using SPSS Statistics (Version 21.0, IBM Corp., New York, NY, USA), Statistix 10 (Analytical Software, Tallahassee, FL, USA), and SuperSMITH Weibull 4.0k-32 software (Wes Fulton, Torrance, CA, USA). Normality was assessed using the Shapiro-Wilk test. F_initial_ values were not normally distributed (*p* < 0.05), whereas F_max_ values showed a normal distribution (*p* > 0.05).

A two-way analysis of variance (ANOVA) was used to analyze the effects of material, substrate, and their interaction on F_max_. Tukey’s HSD post-hoc test was applied for multiple pairwise comparisons (α = 0.05).

Pearson’s correlation analysis was used to explore potential linear associations between bonded surface area and load-to-fracture outcomes (F_initial_ and F_max_). The Pearson`s correlation coefficient (r) ranges from + 1, indicating a perfect positive linear correlation, to − 1, indicating a perfect negative linear correlation, with 0 representing the absence of a linear relationship. The strength of the correlation was interpreted according to Cohen’s classification, with values between 0.10 and 0.29 considered weak, 0.30 to 0.49 moderate, and ≥ 0.50 strong. In addition, the coefficient of determination (R²) was calculated to quantify the proportion of variance in fracture outcomes explained by variations in bonded surface area, providing complementary information on the goodness of fit of the linear model. All correlation analyses were considered exploratory.

Additionally, Weibull analysis was performed for each material-substrate combination to evaluate the distribution of fracture loads and the reliability characteristics of the tested restorations.

## Results

During thermo-mechanical aging, adhesive debonding without visible cracking was observed in a limited number of specimens from the lithium disilicate and resin-matrix composite groups. Specifically, failures occurred in 3 out of 30 specimens in the LDE group, 7 out of 30 specimens in the LDG group, and 3 out of 30 specimens in the RMC group, whereas all zirconia (ZIR) specimens withstood the aging protocol without debonding. These failures may be considered clinically relevant, as they indicate a loss of adhesion under simulated oral service conditions and suggest a greater susceptibility of the LDG configuration to aging-induced interfacial degradation. Specimens that failed during aging were excluded from the load-to-failure analysis, resulting in a maximum reduction of two specimens per subgroup. Statistical analyses were performed using the actual sample size available in each subgroup, and homogeneity of variances was confirmed prior to inferential testing.

The maximum fracture load (F_max_) varied across the different material-substrate combinations (Table 3). The two-way ANOVA revealed statistically significant main effects for both material (F = 12.18, *p* < 0.001) and substrate (F = 12.96, *p* < 0.001). In addition, a significant interaction effect between material and substrate was detected (F = 4.10, *p* = 0.001).

**Table 3:** Mean (N), standard deviation (SD), and Weibull modulus (*m*) for the monotonic load-to-fracture test

Material	Substrate	F_initial_	F_max_	
		Mean ± SD	Mean ± SD*	Weibull Modulus (95% CI)**
LDE	DE	2607 ± 900	3020 ± 901^ABCD^	2.57^AB^ (1.50–3.89)
	D	1793 ± 483	2161 ± 581^CD^	1.08^B^ (0.61–1.68)
	C	2970 ± 889	4349 ± 1520^A^	1.64^AB^ (0.89–2.64)
LDG	DE	1751 ± 391	2309 ± 1343^BCD^	1.17^B^ (0.65–1.87)
	D	1075 ± 361	1081 ± 684^D^	1.46^AB^ (0.69–2.57)
	C	2785 ± 781	4049 ± 1375^AB^	1.56^AB^ (0.84–2.54)
RMC	DE	2600 ± 631	3084 ± 652 ^ABCD^	2.09^AB^ (1.21–3.24)
	D	1741 ± 1025	2245 ± 1071^BCD^	1.84^AB^ (1.00–2.99)
	C	2823 ± 1254	3627 ± 1838^ABC^	2.63^AB^ (1.43–4.24)
ZIR	DE	3422 ± 1477	4577 ± 1255^A^	3.87^A^ (2.16–6.15)
	D	2469 ± 601	4514 ± 923^A^	2.81^AB^ (1.66–4.21)
	C	2971 ± 671	3888 ± 873^ABC^	3.18^A^ (1.88–4.76)

* Different letters indicate statistical differences, as determined by Two-way ANOVA and Tukey’s post-hoc tests (α= 0.05)

** Different letters indicate statistical differences according to Weibull analysis using the maximum likelihood estimation method, with overlapping 95% confidence intervals indicating statistical similarities

For lithium disilicate IPS e.max CAD (LDE), the highest mean F_max_ (N) was observed for restorations bonded to resin composite (C) (4349 ± 1520), followed by those bonded to dentin surrounded by enamel (DE) (3020 ± 901), whereas the lowest values were recorded for dentin (D) (2161 ± 581). A similar trend was observed for lithium disilicate Initial LiSi Block (LDG), with the highest mean F_max_ for LDG-C (4049 ± 1375) and markedly lower values for LDG-DE (2309 ± 1343) and LDG-D (1081 ± 684).

The resin-matrix composite (RMC) groups exhibited a more homogeneous performance across substrates, although a gradual decrease in F_max_ was still evident from C (3627 ± 1838) to DE (3084 ± 652) and D (2245 ± 1071). In contrast, zirconia (ZIR) restorations showed consistently high fracture loads across all substrate conditions, with mean F_max_ values of 4577 ± 1255 for ZIR-DE, 4514 ± 923 for ZIR-D, and 3888 ± 873 for ZIR-C, without a clear substrate-dependent trend.

**Fig. 3:**
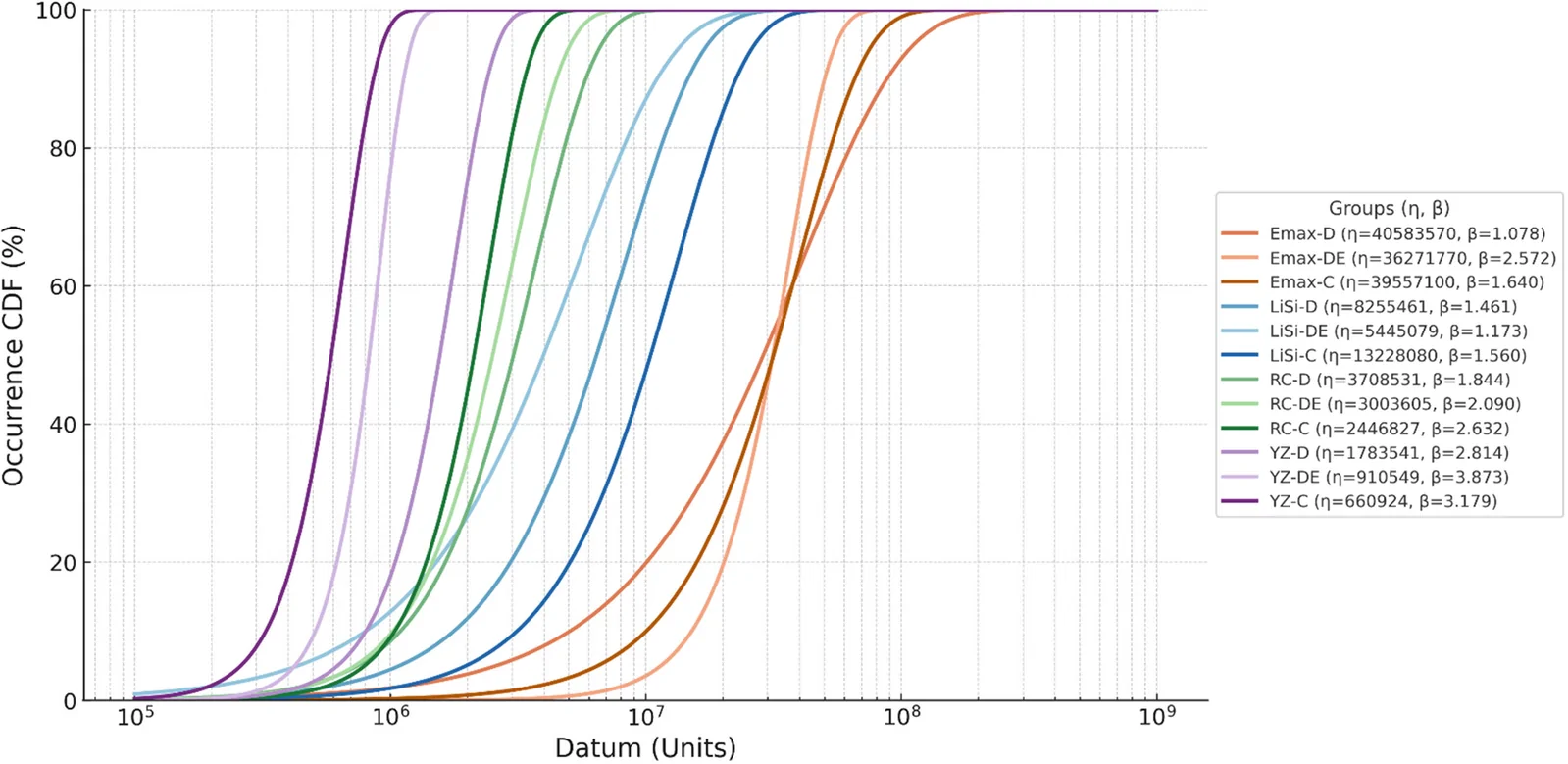
Weibull probability plots showing the cumulative distribution function (CDF) of monotonic load-to-fracture for all substrate-material combinations. The plot compares the different groups on the estimated characteristic strength (η) and Weibull modulus (β)

The Weibull analysis (Fig. 3) demonstrated marked differences in the reliability of load-bearing performance among the tested groups (Table 4). ZIR bonded to dentin surrounded by enamel (ZIR-DE) exhibited the highest Weibull modulus (m = 3.87; 95% CI = 2.16–6.15), followed by ZIR bonded to resin composite (ZIR-C) (m = 3.18; CI = 1.88–4.76), indicating a narrow distribution of failure loads and high structural reliability.

**Table 4:** Pearson’s correlation coefficients (r) describing the linear association between bonded surface area and load-to-fracture outcomes (F_initial_ and F_max_) for each material and substrate condition

	Correlations (*r*)					
	F_initial_			F_max_		
	DE	D	C	DE	D	C
LDE	0.477	-0.190	-0.529	0.661	-0.218	0.224
LDG	0.206	0.656	0.423	-0.567	-0.349	0.200
RMC	0.203	-0.253	0.094	0.107	-0.308	0.272
ZIR	-0.500	0.080	-0.138	-0.336	0.103	-0.535

In contrast, the lowest Weibull moduli were observed for LDE bonded to dentin (LDE-D) (m = 1.08; CI = 0.61–1.68) and LDG bonded to dentin surrounded by enamel (LDG-DE) (m = 1.17; CI = 0.65–1.87), reflecting greater variability in failure loads. All remaining material-substrate combinations showed intermediate Weibull moduli, with values that were statistically similar to each other and to those of the highest or lowest reliability groups.

**Fig. 4:**
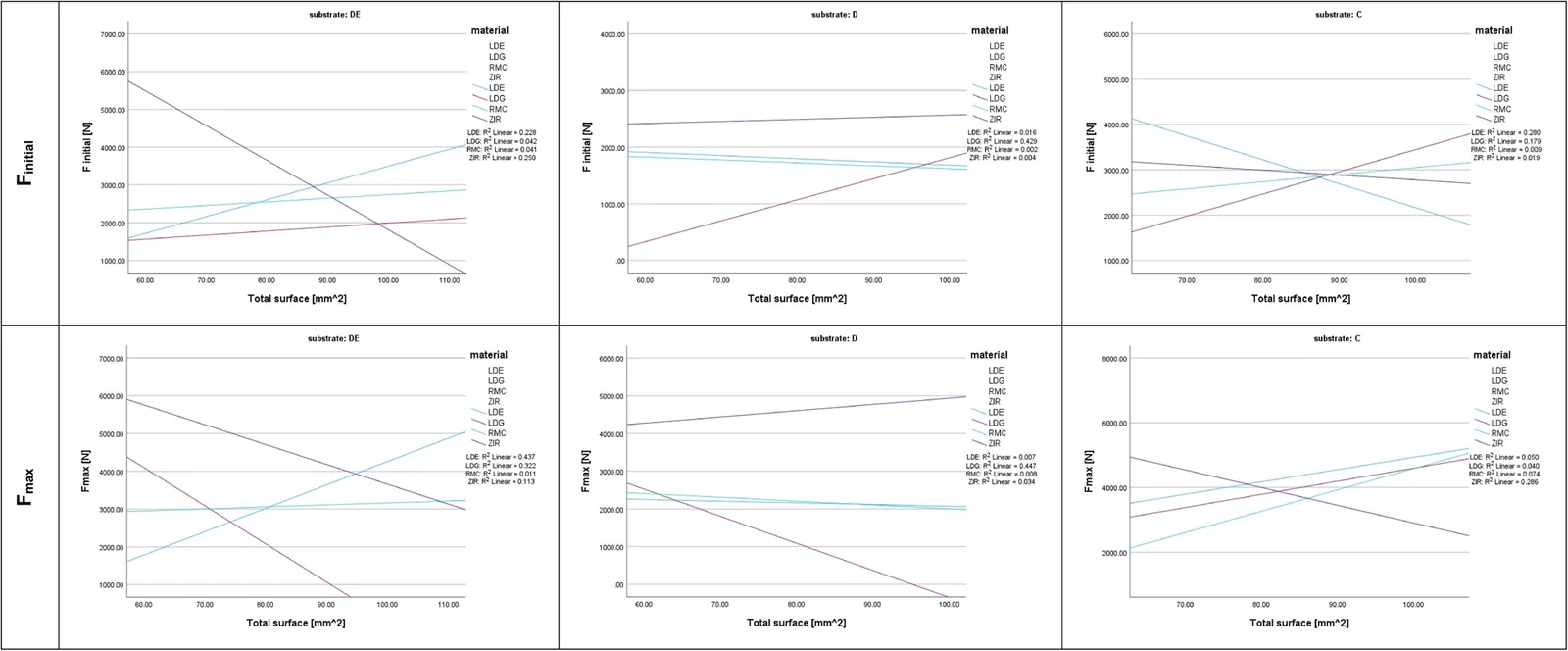
Coefficient of determination (R²) describing the proportion of variance in load-to-fracture outcomes (F_initial_ and F_max_) explained by bonded surface area for each material-substrate combination

The Pearson’s correlation analysis (Table 4, Fig. 4) revealed material- and substrate-dependent trends between bonded surface area and initial crack formation force (F_initial_). For the dentin-enamel (DE) substrate, positive correlations were observed for LDE (*r* = 0.48), LDG (*r* = 0.21) and RMC (*r* = 0.20), whereas ZIR exhibited a moderate negative correlation (*r*=-0.50). For specimens bonded to composite substrate (C), LDE showed a negative correlation (*r* = − 0.53), while LDG demonstrated a positive correlation (*r* = 0.42), RMC showed a negligible positive correlation (*r* = 0.10), and ZIR exhibited a weak negative correlation (*r*=-0.14). For the dentin (D) substrate, weak negative correlations were found for LDE (*r*=-0.19) and RMC (*r*=-0.25), whereas LDG showed a strong positive correlation (*r* = 0.66) and ZIR a negligible positive correlation (*r* = 0.08).

Regarding the relationship between bonded surface area and F_max_, moderate and weak correlations were observed depending on the material and substrate. For the DE substrate, a moderate positive correlation was found for LDE (*r* = 0.66), whereas LDG (*r*=-0.57) and ZIR (*r*=-0.34) showed negative correlations. RMC presented a negligible positive correlation (*r* = 0.10). For the C substrate, weak positive correlations were observed for LDE (*r* = 0.22), LDG (*r* = 0.20), and RMC (*r* = 0.27), while ZIR again showed a negative correlation (*r*=-0.53). For the D substrate, weak negative correlations were identified for LDE (*r*=-0.22), LDG (*r*=-0.35), and RMC (*r*=-0.31), while ZIR demonstrated a negligible positive correlation (*r* = 0.10).

**Fig. 5:**
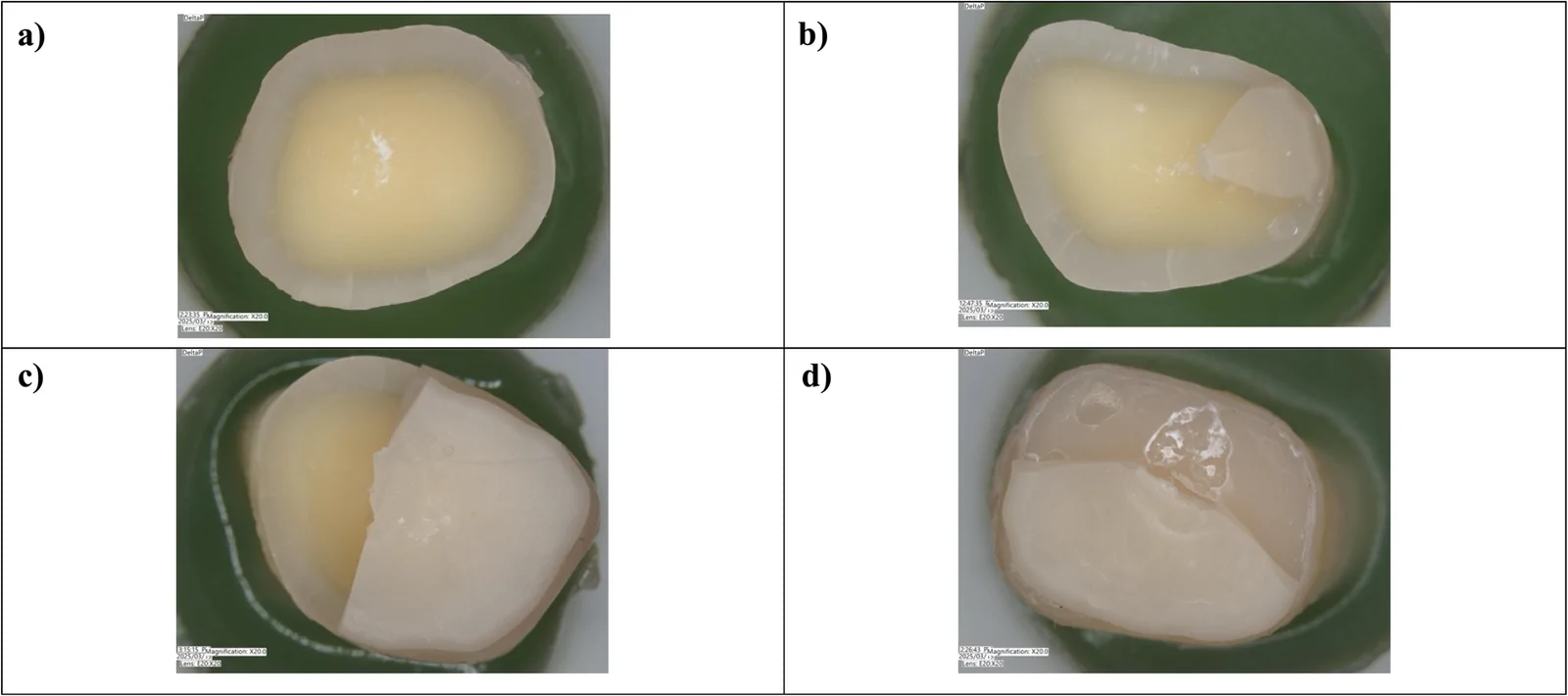
Images **a**, **b**, **c**, and **d** present examples of the different failure types: (**a**) Type 0 (adhesive substrate failure) and Type A (adhesive veneer failure); (**b**) Type 1 (mixed substrate failure) and Type B (cohesive veneer failure); (**c**) Type 2 (mixed substrate failure) and Type B; (**d**) Type 3 (cohesive substrate failure) and Type B

Across all materials and substrate conditions, substrate-related failures were predominantly adhesive or mixed in nature (Fig. 5). Type 0 (adhesive failure) and Type 1 (mixed adhesive/cohesive failure) accounted for the majority of substrate failures, particularly for lithium disilicate materials (LDE and LDG), regardless of the bonded substrate. In contrast, Type 3 (cohesive substrate failure) was more frequently observed for RMC and ZIR, especially when bonded to dentin or dentin surrounded by enamel, indicating a higher tendency for cohesive damage within the substrate for these material-substrate combinations.

Regarding the occlusal veneer failure modes, Type B (cohesive failure) was consistently the most prevalent failure type across all materials and substrates, with proportions ranging from 60 to 100% (Fig. 5). Cohesive veneer failures were particularly dominant for zirconia restorations, which exhibited 100% Type B failures when bonded to dentin and resin composite substrates. Type A (adhesive veneer failure) occurred less frequently and was mainly observed in lithium disilicate and resin-matrix composite groups, especially when bonded to dentin-enamel substrates. The distribution of failure modes for each material-substrate combination is presented in detail in Table 5.

**Table 5:** Failure types. Substrate failure: Type 0 – adhesive failure; Type 1 – mixed failure (adhesive and cohesive) with < 1/3 remnant; Type 2 – mixed failure with > 2/3 remnant; Type 3 – cohesive failure. Occlusal veneer failure: Type A – adhesive failure; Type B – cohesive failure

Material	Substrate	Substrate Failure Type				Occlusal Veneer Failure Type	
		Type 0 (%)	Type 1 (%)	Type 2 (%)	Type 3 (%)	Type A (%)	Type B (%)
LDE	DE	40	50	0	10	30	70
	D	40	60	0	0	30	70
	C	50	12	25	12	25	75
LDG	DE	30	70	0	0	30	70
	D	11	22	33	33	22	78
	C	40	20	30	10	10	90
RMC	DE	30	30	30	10	40	60
	D	20	20	10	50	30	70
	C	33	11	33	22	33	67
ZIR	DE	33	11	0	55	33	67
	D	40	30	30	0	0	100
	C	20	20	60	0	0	100

## Discussion

The present study investigated the influence of substrate condition and restorative material on the load-bearing capacity of ultra-thin occlusal veneers. Based on the results obtained, both null hypotheses were rejected, as both substrate type and restorative material significantly affected F_max_. The significant interaction observed between these factors indicates that the mechanical behavior of minimally invasive occlusal veneers is governed by the combined effect of material properties and the quality of the bonded substrate.

Substrate condition emerged as a key determinant of mechanical performance for most materials evaluated. For lithium disilicate (LDE and LDG), restorations bonded exclusively to dentin (D) consistently exhibited the lowest load-bearing capacity, whereas bonding to resin composite (C) resulted in the highest F_max_ values, with dentin surrounded by enamel (DE) showing intermediate performance. Notably, the LDG groups exhibited the highest incidence of adhesive debonding during thermo-mechanical aging. Although these failures occurred before load-to-fracture testing and involved a limited number of specimens, they may indicate greater susceptibility of the bonded interface to aging-induced degradation under simulated oral conditions. This trend was confirmed by Weibull analysis, which revealed reduced reliability for dentin-bonded lithium disilicate groups, particularly LDE-D, and improved reliability when enamel or resin composite was present. These findings align with previous reports demonstrating that adhesion to dentin is inherently less reliable than adhesion to enamel, due to dentin’s higher organic content, tubular morphology, intrinsic moisture, and increased susceptibility to hydrolytic degradation of the adhesive interface [25]. In contrast, enamel provides a more homogeneous and highly mineralized substrate, enabling more effective micromechanical interlocking and long-term bond stability, which has been associated with improved mechanical performance of occlusal veneers [8, 9, 12].

The presence of circumferential enamel margins likely increased the proportion of enamel available for bonding, thereby enhancing both load-bearing capacity and reliability, as also suggested by previous studies evaluating minimally invasive restorations bonded to enamel-containing substrates [9, 13]. Similarly, bonding to a resin composite substrate provided a standardized and mechanically favourable bonding surface, which may explain the consistently higher F_max_ values observed for the C substrate. From a biomechanical standpoint, the elastic modulus of resin composite, which is closer to that of dentin, may contribute to improved stress distribution at the adhesive interface [19, 21].

In this study, distinct material-dependent behaviors were observed. Zirconia restorations exhibited consistently high fracture loads and reliability across all substrate conditions, with no statistically significant differences between dentin, dentin surrounded by enamel, and resin composite. This substrate-independent performance is attributed to zirconia’s superior intrinsic mechanical properties, such as high fracture toughness and flexural strength [16, 17]. Similar findings have been reported for ultra-thin zirconia occlusal veneers subjected to fatigue and fracture testing [10, 15], where zirconia also demonstrated high fracture resistance and lower sensitivity to substrate-related variations. This behavior reinforces the concept that the mechanical performance of zirconia restorations may be governed more by intrinsic material properties than by adhesive factors. In contrast, lithium disilicate restorations were clearly substrate dependent. Both lithium disilicate materials showed reduced load-bearing capacity when bonded exclusively to dentin compared with resin composite substrates, confirming the strong reliance of glass-ceramics on a stable adhesive interface under minimal thickness conditions [8, 25]. Similar substrate-dependent behavior has been reported in previous studies evaluating ultra-thin occlusal veneers, in which the preservation of enamel and the quality of the bonding substrate significantly influenced restoration performance and reliability [8, 9, 12, 13]. The resin-matrix composite showed an intermediate and more homogeneous response across substrates. This behavior may be related to its elastic modulus being closer to dentin, promoting more favourable stress distribution and reducing stress concentration at the adhesive interface [19, 20].

Failure mode analysis provided additional insight into the biomechanical behavior of the restorative systems. Lithium disilicate groups exhibited a higher incidence of adhesive and mixed failures, particularly when bonded to dentin, suggesting a stronger dependence on the integrity of the adhesive interface. In contrast, zirconia restorations predominantly exhibited cohesive failures, indicating that the adhesive interface remained relatively stable and that stresses were more frequently transferred to the restoration or substrate itself. This behavior may be associated with zirconia’s high elastic modulus, fracture toughness, and flexural strength, which contribute to greater resistance to deformation and interfacial failure [16, 17]. Resin-matrix composite restorations showed an intermediate behavior, with failure patterns reflecting a more balanced stress distribution between the restoration, adhesive interface, and substrate. This may be related to the elastic modulus of resin-matrix composite, which is closer to that of dentin and may reduce stress concentration at the adhesive interface, thereby explaining the more homogeneous mechanical response observed across substrates [19, 20]. Clinically, these findings suggest that lithium disilicate restorations require careful substrate management and adhesive procedures, whereas zirconia may offer greater predictability in situations where substrate conditions are less favorable. Resin-matrix composite may represent a biomechanically favourable alternative when stress modulation and compatibility with dentin-like substrates are desired.

The correlation analyses revealed material- and substrate-dependent relationships between bonded surface area and both Finitial and Fmax. Positive correlations were observed for some material-substrate combinations, particularly for lithium disilicate and resin-matrix composite restorations bonded to dentin-enamel substrates, suggesting that a greater bonded surface area may contribute to enhanced crack initiation resistance and load-bearing capacity under specific conditions. However, this trend was not consistently observed across all groups, as negative or weak correlations were also identified for certain combinations, including LDE bonded to composite substrate and several zirconia groups. These findings suggest that the influence of bonded surface area on mechanical performance is not universal and may also depend on material properties and stress distribution patterns. Previous studies have similarly suggested that adhesive surface extent and internal adaptation can influence the mechanical behavior of minimally invasive restorations [11]. In contrast, zirconia frequently exhibited weak or negative correlations, reinforcing the notion that its mechanical performance is predominantly governed by intrinsic material properties rather than adhesive surface area.

From a clinical perspective, the present findings indicate that substrate management is as critical as material selection for the performance of minimally invasive occlusal veneers. Preserving enamel whenever possible, or alternatively creating a resin composite substrate, may enhance the predictability of restorations, particularly when lithium disilicate is used, given its greater dependence on a stable adhesive interface. In situations where optimal bonding conditions are limited, zirconia may provide a more predictable mechanical response because of its lower sensitivity to substrate-related variations. Therefore, careful evaluation of the remaining tooth structure and substrate condition should be considered during treatment planning for minimally invasive occlusal restorations. It should be noted, however, that this study was conducted in vitro, and although the applied thermo-mechanical aging protocol simulates long-term masticatory loading, it cannot fully reproduce the complexity of the oral environment. In addition, only one restoration thickness and one adhesive strategy per material were evaluated. Other methodological limitations should also be considered, including the absence of surface characterization analyses and internal fit and marginal adaptation assessments, and lack of direct verification of cement thickness, which may influence the biomechanical behavior of the restorations. Furthermore, the use of natural teeth may introduce intrinsic anatomical variability despite standardization procedures, and the circumferential enamel margins were not quantitatively standardized. Future studies should therefore explore different restoration thicknesses and bonding approaches, incorporate quantitative characterization methods, and validate these findings under clinical conditions.

## Conclusions

From this study, the following can be concluded:

1. Both substrate condition and restorative material significantly affected the load-bearing capacity of ultra-thin occlusal veneers.
2. Bonding exclusively to dentin resulted in reduced load-bearing capacity and reliability for lithium disilicate restorations, whereas the presence of surrounding enamel or a resin composite substrate improved mechanical performance.
3. Zirconia restorations exhibited consistently high fracture loads and reliability regardless of substrate condition, indicating lower sensitivity to variations in the bonded substrate compared with lithium disilicate.
4. Resin-matrix composite restorations demonstrated intermediate mechanical behaviour, with more homogeneous performance across substrates, suggesting potential advantages in stress distribution and reparability.
5. The influence of bonded surface area on crack resistance and load-bearing behavior appeared to be material- and substrate-dependent, with positive associations observed only for specific restorative material-substrate combinations.

## Data Availability

The datasets generated and/or analyzed during the current study are available from the authors on reasonable request.
